# Hypertrophic Cardiomyopathy: New Clinical and Therapeutic Perspectives of an “Old” Genetic Myocardial Disease

**DOI:** 10.3390/genes16010074

**Published:** 2025-01-10

**Authors:** Chiara Calore, Mario Mangia, Cristina Basso, Domenico Corrado, Gaetano Thiene

**Affiliations:** Department of Cardiac, Thoracic, Vascular Sciences and Public Health, University of Padua Medical School, 35128 Padova, Italy; mario.mangia9@gmail.com (M.M.); cristina.basso@unipd.it (C.B.); domenico.corrado@unipd.it (D.C.); gaetano.thiene@unipd.it (G.T.)

**Keywords:** hypertrophic cardiomyopathy, genetics, sudden cardiac death, heart failure, treatment, new drugs, surgery, transplantation

## Abstract

Since its first pathological description over 65 years ago, hypertrophic cardiomyopathy (HCM), with a worldwide prevalence of 1:500, has emerged as the most common genetically determined cardiac disease. Diagnostic work-up has dramatically improved over the last decades, from clinical suspicion and abnormal electrocardiographic findings to hemodynamic studies, echocardiography, contrast-enhanced cardiac magnetic resonance, and genetic testing. The implementation of screening programs and the use of implantable cardioverter defibrillators (ICDs) for high-risk individuals have notably reduced arrhythmic sudden deaths, altering the disease’s mortality profile. Therapeutic breakthroughs, including surgical myectomy, alcohol septal ablation, and the novel introduction of “myosin inhibitors”, have revolutionized symptom management and reduced progression to advanced heart failure (HF) and death. Despite this progress, refractory HF—both with preserved and reduced systolic function—has become the predominant cause of HCM-related mortality. While most patients with HCM experience a favorable clinical course with low morbidity and mortality, timely identification and targeted treatment of high-risk subgroups progressing toward progressive HF remain a pressing challenge, even for expert clinicians.

## 1. Introduction

Over six decades have passed since hypertrophic cardiomyopathy (HCM) was first described by Donald Teare in 1958 and defined as idiopathic hypertrophic subaortic stenosis by Braunwald and Morrow [1,2,3,4,5]. These seminal contributions established the foundation for HCM’s recognition as a family disorder with an estimated prevalence of 1:500 in the general population.

The genetic underpinnings of HCM were elucidated beginning in 1990 with the identification of a missense mutation in the β-cardiac myosin heavy chain gene [6,7]. Since then, over 1300 mutations across 13 sarcomeric and sarcomere-associated genes have been discovered, defining HCM as a genetic sarcomeric disorder [8]. Advances in imaging techniques, particularly cardiac magnetic resonance (CMR), have enhanced the evaluation of hypertrophy patterns, mitral valve anomalies, and myocardial tissue characteristics [9]. With its exclusive ability for tissue characterization, including T1 and T2 mapping sequences and extracellular volume estimation, CMR has also demonstrated good accuracy in the differential diagnosis with other forms of hypertrophy, such as amyloidosis and Fabry disease [10]. Moreover, CMR’s ability to detect late gadolinium enhancement (LGE) has furthered its role in sudden cardiac death risk stratification [11,12].

**Figure 1:**
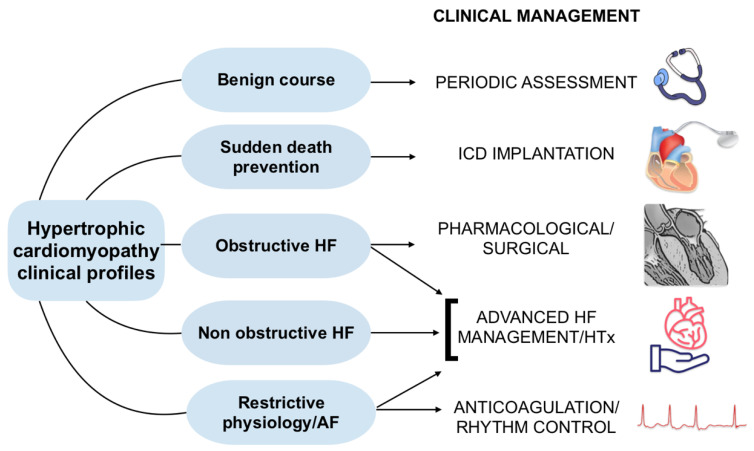
Main prognostic pathways in HCM.

Historically associated with high sudden death rates among young individuals, HCM management has transformed, with population-wide screening programs, such as the Italian one for competitive athletes, proving effective in early detection and event prevention [13,14]. Contemporary management strategies have dramatically decreased HCM-related mortality, reducing rates from approximately 6% annually to less than 0.5% [15]. Five prognostic pathways illustrate the diverse clinical courses of HCM, encompassing arrhythmic risks and progression to heart failure (HF) in obstructive and non-obstructive forms (Figure 1). While over 80% of patients achieve normal longevity and a good quality of life, a minority require specialized interventions to address severe complications [15,16,17].

## 2. Sudden Death Prevention

**Figure 2:**
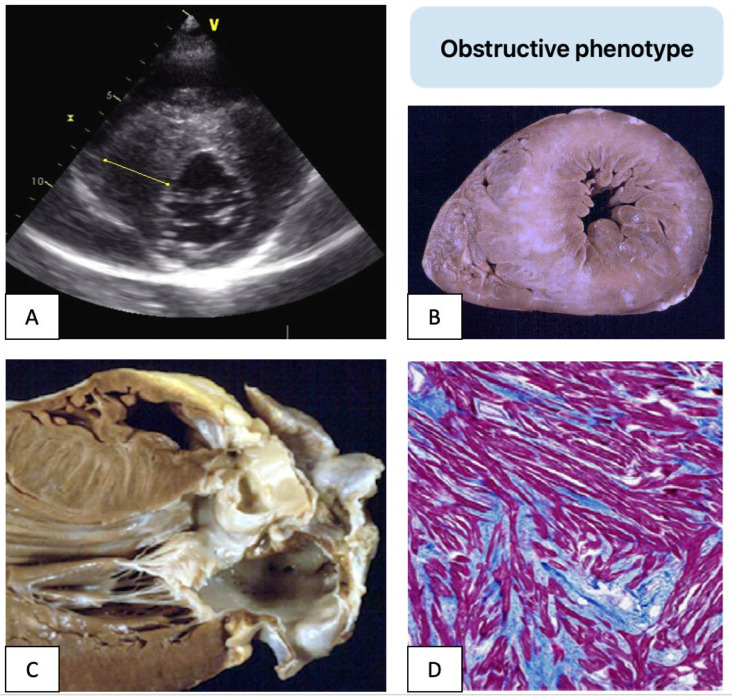
Sudden death in obstructive HCM. Features of a young HCM patient who died suddenly. Echocardiography short-axis view shows severe asymmetric left ventricular hypertrophy (**A**) and pathological postmortem examination reveals macroscopic fibrosis, subaortic septal bulging, endocardial fibrous plaque, and a thickened anterior mitral valve (**B**,**C**). Histological section highlights myocardial disarray with interstitial and replacement fibrosis (Heidenhain’s Trichrome stain) (**D**).

Beyond the dynamic obstruction characteristic of HCM, myocardial ischemia and fibrotic scars have been identified as key substrates for ventricular arrhythmias and sudden cardiac death [18]. Postmortem examinations and advanced imaging modalities, such as CMR, have revealed macroscopic fibrosis and myocardial disarray as major contributors to arrhythmic risk (Figure 2).

The introduction of implantable cardioverter defibrillators (ICDs) in the late 1990s represented a transformative step in the prevention of arrhythmic deaths. ICDs have demonstrated efficacy in terminating life-threatening ventricular tachycardia, with intervention rates of 3–4% annually for primary prevention and up to 10% for secondary prevention following resuscitated cardiac arrest [19]. Despite this success, risk stratification for ICD implantation remains a significant challenge due to the unpredictable timing of arrhythmic events.

Several predictive markers have been identified, forming the basis for decision-making algorithms endorsed by leading guidelines. Major risk factors, as outlined by the American Heart Association (AHA) and the American College of Cardiology (ACC), include extreme left ventricular hypertrophy (>30 mm), recent unexplained syncope, a family history of sudden cardiac death, and non-sustained ventricular tachycardia detected during ambulatory monitoring [20]. More recently, markers such as extensive LGE on CMR, progression to an end-stage phenotype with reduced ejection fraction (<50%), and the presence of left ventricular apical aneurysms have been integrated into clinical practice [21,22].

While the AHA/ACC strategy prioritizes sensitivity to identify high-risk patients [16], the European Society of Cardiology (ESC) has developed a multivariable risk score to refine specificity and reduce unnecessary ICD placements [23,24]. The ESC calculator incorporates seven clinical variables into a logistic regression model to estimate individual risk. These complementary approaches allow clinicians to tailor risk assessment based on patient-specific factors, balancing the benefits of prevention against the risks of device-related complications.

Subcutaneous ICDs have emerged as a viable alternative to traditional transvenous systems, particularly in younger patients without pacing requirements. These devices minimize the risk of infection and mechanical complications but require careful patient selection to avoid issues like T-wave oversensing [25]. Transparent communication with patients regarding the benefits and limitations of ICD therapy remains crucial, ensuring informed decision-making and alignment with patient preferences.

## 3. Heart Failure Pathways

As the incidence of arrhythmic deaths has significantly declined, advanced HF has emerged as the predominant adverse outcome in HCM [22]. This complication, responsible for approximately two-thirds of HCM-related deaths, presents in both obstructive and non-obstructive forms, with varying mechanisms and clinical profiles [26,27,28].

Left ventricular (LV) outflow obstruction is a major contributor to HF symptoms, present in up to 70% of patients. Subaortic or intracavitary gradients lead to exertional dyspnea, fatigue, chest pain, and syncope, which progressively worsen without intervention. Surgical myectomy and alcohol septal ablation remain effective treatments, alleviating symptoms and reducing obstruction-related HF progression. In non-obstructive HCM, a smaller subset of patients (5–10%) develop refractory HF (NYHA class III–IV) despite preserved systolic function, necessitating heart transplantation as a definitive therapy [26].

**Figure 3:**
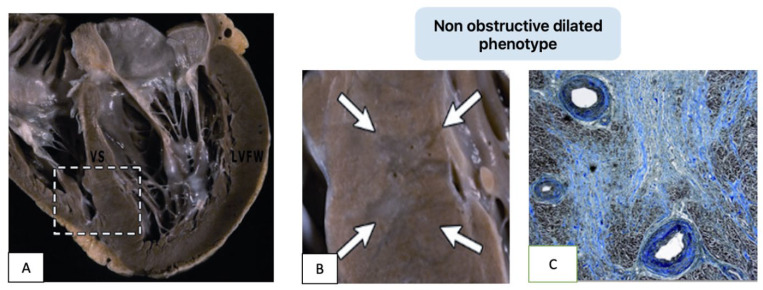
“End-stage” evolution of HCM with dilated LV and systolic dysfunction. Heart of a male HCM patient removed at transplantation. Gross examination reveals thinning of the basal and midventricular septum with respect to apical portion (**A**); a large septal scar is highlighted by arrows (**B**). Histological section of the septum (**C**) demonstrates extensive replacement fibrosis with abnormal intramural arterioles (Trichrome stain).

The progression to advanced HF often involves a transition from a hyperdynamic, nondilated LV to one with systolic dysfunction (ejection fraction < 50%). This “end-stage” phenotype is characterized by adverse remodeling, chamber dilation, and diffuse myocardial fibrosis, resembling dilated cardiomyopathy (Figure 3).

Guideline-directed pharmacological therapies, resynchronization devices, and ICDs are critical in managing these patients. Despite the severe phenotype, contemporary treatments have improved outcomes in this subset of patients, with 10-year survival rates reaching 85% with or without transplantation [27].

**Figure 4:**
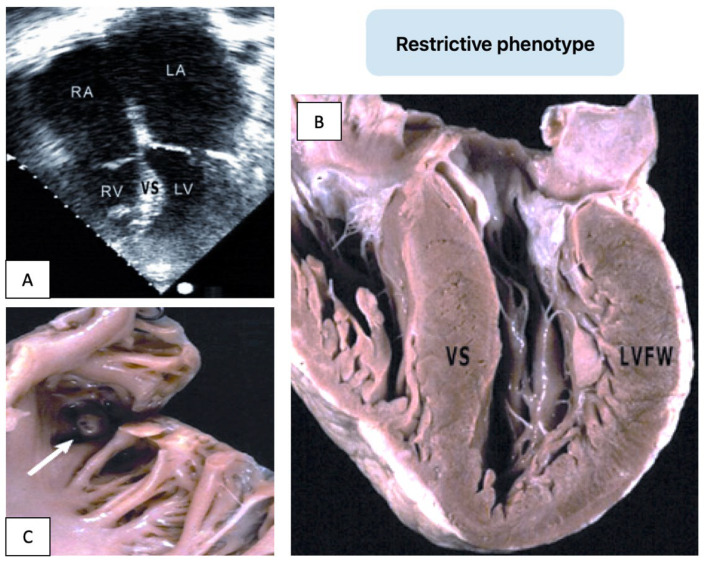
Heart failure in HCM secondary to restrictive physiology and atrial fibrillation. Echocardiographic 4-chamber view (**A**) and gross pathology findings (**B**,**C**) from a male patient with sarcomeric hypertrophic cardiomyopathy (β-myosin heavy chain mutation). Severe dilation of the right (RA) and left atria (LA), normal-sized left (LV) and right ventricles (RV), and mild asymmetric hypertrophy of the ventricular septum (VS) compared to the left ventricular free wall (LVFW) are observed. A thrombus within the LA appendage is indicated by an arrow.

Another HF pathway in HCM is characterized by preserved systolic function but severe diastolic dysfunction, known as “restrictive physiology” [28]. This form, associated with mild hypertrophy and small ventricular cavities, results in increased filling pressures, marked atrial dilation, and frequent atrial fibrillation (AF). Symptoms include reduced exercise tolerance, congestive HF, and embolic events due to atrial thrombi (Figure 4).

**Figure 5:**
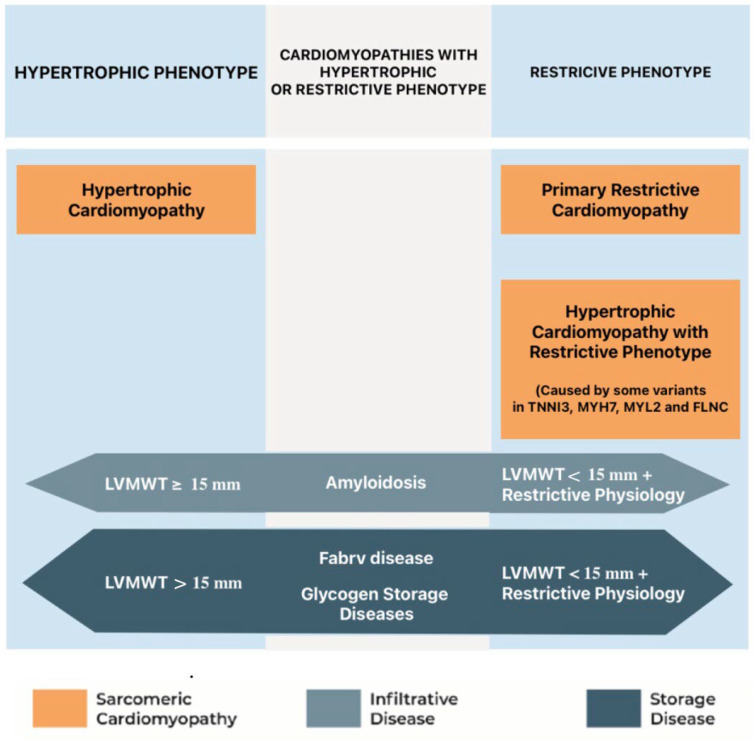
Comparison between HCM, RCM phenotypes, and phenocopies. LVMWT: left ventricular maximal wall thickness. With permission from Vio et al. [29].

Differentiating HCM with restrictive physiology from primary restrictive cardiomyopathies (RCMs) or phenocopies can be challenging due to overlapping features but is essential for appropriate management ([29], Figure 5).

In 2007, Kubo was the first to describe the “restrictive phenotype” in a family with troponin I mutation as an uncommon presentation of HCM (about 1.5% of cases), associated with severe functional limitation and poor prognosis, resembling idiopathic RCM [30,31]. Recent studies suggest that genetic and environmental modifiers may influence the progression to restrictive or end-stage phenotypes. Sarcomeric mutations, particularly in myosin heavy chain (*MYH7*), cardiac troponins (*TNNI3*, *TNNT2*), α cardiac actin (*ACTC*), myosin binding protein C (*MYBPC3*), tropomyosin 1 (*TPM1*), and myosin regulatory light chain (*MYL2*), are common in these forms. Filamin C (*FLNC*), a muscle-specific protein involved in myocyte differentiation and interaction with proteins of both the Z-disc and the sarcolemma, was initially described as a genetic substrate for various forms of hereditary peripheral myopathies. In recent years, mutations of *FLNC* have been linked to different forms of genetic cardiomyopathies including dilated, arrhythmogenic, hypertrophic, and restrictive cardiomyopathy [32,33,34]. Genotype–phenotype correlation in *FLNC*-related cardiomyopathies is highly dependent on the type and site of mutation [33]. Truncating *FLNC* mutations typically manifest as dilated or arrhythmogenic cardiomyopathy, whereas *FLNC*-related HCM or RCM phenotypes are usually linked to missense mutations. Although *FLNC* variants account for only 1.3–2% of mutation-positive HCM cases, affected individuals frequently present with severe phenotypes and poor clinical outcomes. Recently, a novel pathogenic *FLNC* variant has been identified in a large French-Canadian family resulting in a severe phenotype of a mixed hypertrophic–restrictive cardiomyopathy [35]. Given the common genetic basis, the term “sarcomyopathies” was subsequently used to include both HCM and RCM forms [36,37,38,39,40,41]. Although, in these forms, genetic data are important for a correct diagnosis and classification of the disease, to date, they do not influence the therapeutic choice. It will be interesting in the future to evaluate whether particular mutations influence the response to specific therapies, such as myosin inhibitors, or whether drugs aimed at improving diastolic function may be particularly indicated in these forms. Comprehensive evaluation, including advanced imaging and genetic testing, is crucial for early identification and timely intervention, potentially altering the clinical course for high-risk patients who present a particularly malignant prognosis, with earlier symptom onset, rapid progression to advanced heart failure, and transplantation as the only currently available therapeutic option [28].

## 4. Pharmacological Therapy

In April 1964, leading cardiologists, cardiovascular surgeons, and pathologists convened in London to discuss obstructive HCM, a condition then recognized as a relatively new disease entity. Discussions focused on invasive hemodynamic studies conducted between 1960 and 1964, which demonstrated that dynamic left ventricular outflow tract (LVOT) obstruction is exacerbated by increases in cardiac contractility, decreases in afterload, and reductions in preload [42].

In 2024, these foundational principles continued to inform treatment strategies for symptomatic HCM [16,17]. Pharmacological interventions target heart failure symptoms, angina, arrhythmias, and LVOT obstruction. β-blockers (BBs) without vasodilatory properties and nondihydropyridine calcium-channel blockers (CCBs) remain first-line therapies, effectively reducing symptoms by slowing heart rate and increasing left ventricular preload [16,17,22,43]. However, caution is required when administering verapamil in patients with high resting gradients or advanced heart failure [44].

Disopyramide, a Class Ia antiarrhythmic agent with negative inotropic effects, is often added to BB or CCB therapy for refractory symptoms in obstructive HCM [45]. Although associated with anticholinergic side effects, its efficacy and safety have been validated in large registries [46].

Ranolazine, initially approved for chronic angina, was shown to partially reverse cellular abnormalities by inhibiting late Na+ current [47]. However, preliminary results have not been confirmed in large clinical trials; consequently, it may be considered as a useful adjunct to standard treatment in non-obstructive HCM patients with chronic angina [48].

**Figure 6:**
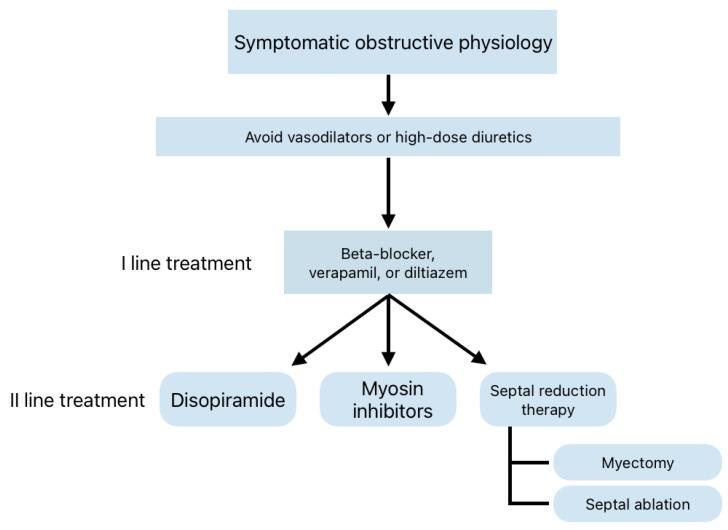
Therapeutic flow-chart according to the most recent guidelines for obstructive HCM (modified from [16]).

Novel therapeutic approaches include cardiac myosin inhibitors, such as mavacamten, which target sarcomeric hypercontractility [49]. The EXPLORER-HCM trial demonstrated significant improvements in LVOT gradients, functional capacity, and biomarker profiles [50]. While a small subset of patients experienced reversible reductions in ejection fraction, long-term studies continue to affirm its safety and efficacy [51]. The VALOR-HCM trial involved adults with symptomatic obstructive HCM who were eligible for septal reduction therapy (SRT) despite maximal medical therapy. The trial showed that after 16 weeks, only 18% of patients on mavacamten were still candidates for or underwent SRT, compared to 77% on placebo [52]. Nowadays, mavacamten is approved by the FDA and EMA and is recommended in more recent guidelines as a second-line therapy when BBs and CCBs are ineffective or poorly tolerated [17], as shown in Figure 6.

Aficamten, a second-generation myosin inhibitor with a shorter half-life, is undergoing clinical evaluation and has shown promising results in a phase 3, double-blind trial (SEQUOIA-HCM), resulting in a significantly greater improvement in peak oxygen uptake from baseline to week 24 (primary end point), as well as reaching all 10 prespecified secondary end points in symptomatic obstructive HCM patients [53].

The use of myosin inhibitors in non-obstructive HCM remains challenging. Phase 3 trials (ODYSSEY-HCM and ACACIA-HCM) were designed to test the effectiveness of mavacamten and aficamten in improving symptoms and functional capacity in this patient population. Long-term studies finally will determine if myosin inhibitors can modify the natural history of HCM, as preclinical evidence suggests they prevent the development of cardiac hypertrophy, myocyte disarray, and myocardial fibrosis in mouse models [49].

**Figure 7:**
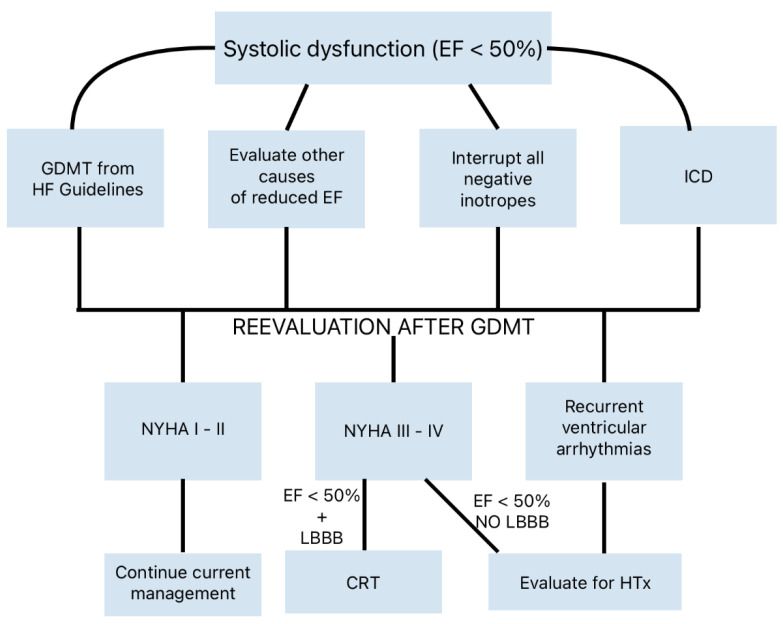
Therapeutic flow-chart according to the most recent guidelines for non-obstructive HCM with systolic dysfunction (modified from [16]). CRT indicates cardiac resynchronization therapy; EF, ejection fraction; GDMT, guideline-directed management and therapy; HTx, heart transplant; ICD, implantable cardioverter defibrillator; LBBB, left bundle branch block; NYHA, New York Heart Association.

Systolic dysfunction (EF < 50%) develops in 5–8% of HCM patients [54]. Medical therapy for HF, including angiotensin receptor–neprilysin inhibitors, ACE inhibitors, ARBs, BBs, mineralocorticoid receptor antagonists, SGLT2 inhibitors, and cardiac resynchronization therapy (CRT), should be considered in a timely manner [16,55]. For refractory HF symptoms, heart transplantation is often the only remaining option (Figure 7).

AF affects about 20% of HCM patients and is associated with a higher risk of thromboembolic complications compared to non-HCM patients and usually requires anticoagulation after the first symptomatic episode, regardless of CHA(2)DS(2)-VASc score. Rhythm control is the preferred strategy, as AF is often poorly tolerated by HCM patients, leading to hemodynamic decompensation and reduced quality of life. Rate control becomes necessary in long-standing disease with severe atrial dilation. Disopyramide may be used for rhythm control, in combination with a rate-controlling agent. Amiodarone, while effective, must be used with caution in young individuals due to its long-term toxicity [56]. Transcatheter ablation is commonly performed early after AF onset but may require multiple procedures and continued use of antiarrhythmic drugs, while biatrial Cox-Maze IV can be performed surgically along with myectomy [57]. The HCM-AF score, a novel risk score created specifically for HCM, can reliably identify patients at high risk for AF [58].

## 5. Interventional/Surgical Therapies

For patients with obstructive HCM and refractory symptoms despite optimal medical therapy, SRTs such as surgical myectomy and alcohol septal ablation (ASA) remain definitive options. Surgical myectomy, particularly in high-volume centers, offers durable relief of LVOT obstruction and is preferred for patients with complex anatomy or concomitant mitral valve disease. The classic Morrow procedure that consists of muscle resection from the basal anterior septum has been improved to an extended septal excision beyond the mitral–septal contact point, including the midventricular septum to the papillary muscles and posterolateral free wall. The complete surgical correction includes mitral valve and submitral structure repair or remodeling to effectively relieve outflow gradient and mitral regurgitation [59]. In patients with AF, concomitant ablation using the Cox-Maze procedure may also be performed [60]. In infants and young children, the modified Konno procedure can serve as an alternative to myectomy when the aortic annulus is too small [60]. In specialized HCM centers, surgery-related mortality has decreased significantly from 6–8% about 30 years ago to approximately 0.5% today [61].

ASA, while effective, carries a higher risk of atrioventricular block (permanent pacemaker implants in 10–15% compared to 1–5% after surgical myectomy) and is generally reserved for patients who are not surgical candidates [62]. While no randomized trials have directly compared surgery and ASA, emerging data suggest that both SRT modalities improve long-term outcomes [63,64,65,66], although a subset of patients progressed to HF, with older age, female sex, and SRT during childhood being associated with a higher risk of developing HF [67].

Advances in alternative techniques, including radiofrequency ablation and transcatheter approaches, may further expand treatment options in the future [68].

In conclusion, after a long history of research and clinical evolution, HCM is now a well understood and highly treatable condition, with low morbidity and mortality. Patients with HCM can expect an acceptable quality of life and nearly normal life expectancy [69].

Although the risk of sudden death has been significantly reduced, and new therapies for gradient reduction in obstructive HCM have been developed, the progression to advanced heart failure with or without systolic dysfunction has emerged as the predominant cause of death in HCM patients. Early identification of those with progressive and refractory symptoms, coupled with appropriate pharmacological or interventional treatments, is essential for improving outcomes in this subset of HCM patients.

## 6. Limitations

This article represents a narrative rather than a systematic review. The objective was to provide a complete and an easy-to-read update on the clinical management of HCM, focused on the most recent discoveries on diagnosis and therapy, without claiming to systematically review all the vast literature on this topic. After a comprehensive evaluation of at least four decades of scientific literature on HCM, we have selected the most recent consensus documents and articles from journals with the highest impact factor, aware that this selection may have potential biases.

## Data Availability

No new data were created or analyzed in this study. Data sharing is not applicable to this article.
